# Fecal metabolomic analysis of the role of gut microbiota and short-chain fatty acids in the therapeutic mechanism of Timosaponin AIII in Sjögren’s syndrome

**DOI:** 10.1007/s10067-024-07294-8

**Published:** 2025-01-18

**Authors:** Fengtao Pang, Quan Jiang, Xiaopo Tang, Kesong Li

**Affiliations:** Department of Rheumatology, Guang’anmen Hospital, Chinese Academy of Chinese Medical Sciences, Beijing, People’s Republic of China

**Keywords:** Gut microbiota, Inflammation, Short-chain fatty acids, Sjogren’s syndrome, Timosaponin AIII

## Abstract

**Introduction/objectives:**

Sjogren’s syndrome (SS) is a chronic inflammatory and difficult-to-treat autoimmune disease. Timosaponin AIII (TAIII), a plant-derived steroidal saponin, effectively inhibits cell proliferation, induces apoptosis, and exhibits anti-inflammatory properties. This study explored the mechanisms of action of TAIII in SS treatment by studying gut microbiota and short-chain fatty acids (SCFAs) using fecal metabolomics.

**Methods:**

The model group used non-obese diabetic (NOD) mice. The treatment group was classified into TAIII and hydroxychloroquine groups. The gut microbiota, SCFAs, and metabolites were analyzed using *16S* rRNA sequencing, gas chromatography–mass spectrometry analysis, and liquid chromatography–mass spectrometry, respectively.

**Results:**

TAIII effectively alleviated dry mouth in NOD mice, slowed the progression of salivary gland tissue injury, reduced inflammatory factor expression, and increased the levels of aquaporins 1 and 5. TAIII regulated SCFA content and tryptophan metabolism by altering the abundance of the Rikenellaceae_RC9_gut_group, thereby reducing the inflammatory response. TAIII can improve imbalances in the gut microbiota and the metabolic levels of related SCFAs and tryptophan, thereby reducing the level of inflammation.

**Conclusion:**

The significant differences observed in the abundance of the Rikenellaceae_RC9_gut_group between the treatment and control groups indicated the potential relationship between bacteria and metabolites in SS.

**Table:** 

**Key Points** • *The safe and effective treatment of SS with traditional Chinese medicine* • *Multi-means study on intestinal flora, short-chain fatty acids, and metabonomics*

**Supplementary Information:**

The online version contains supplementary material available at 10.1007/s10067-024-07294-8.

## Introduction

Sjögren’s syndrome (SS) is a chronic inflammatory autoimmune disease that primarily affects exocrine glands, such as the salivary and lacrimal glands [1]. It mainly affects middle-aged women, with a female to male incidence ratio of approximately 9:1 [2]. The main characteristics include substantially dry mouth and eyes, which may be accompanied by damage to other systems [3]. SS is often accompanied by other rheumatic diseases, can develop into non-Hodgkin lymphoma [4] or lymphocytic leukemia [5] in severe cases, and is difficult to treat. Abnormal activation of T and B cells, particularly by cytokines associated with Th17 cells, such as interleukin (IL)−17 and IL-23, along with inflammatory responses driven by B cell–related factors, such as TNF and BAFF, are key contributors to the development of SS [6, 7].

Timosaponin AIII (TAIII), a type of steroidal saponin derived from the *Anemarrhena asphodeloides* Bunge plant, exhibits a diverse range of pharmacological activities. TAII influences various cellular signaling pathways, through which it exerts anti-platelet aggregation [8], anti-inflammation [9], anti-tumor [10], and blood sugar regulation abilities [11]. TAIII can effectively inhibit cell proliferation, induce apoptosis, suppress cell migration and invasion, and exert anti-inflammatory and anti-angiogenic properties [12].

The gut microflora play a crucial role in maintaining intestinal homeostasis. They substantially influence the regulation of the human digestion and the immune system [13]. Changes in the gut microflora can adversely affect immune system function [14]. Patients with SS exhibit a pronounced disruption in their intestinal microflora; this imbalance considerably affects the severity of dry eyes [15–17] and is correlated with systemic disease activity and gastrointestinal inflammation [18]. Short-chain fatty acids (SCFAs) are metabolites produced by intestinal microorganisms during the fermentation of indigestible carbohydrates. They play crucial roles in maintaining intestinal barrier function, regulating immune responses, facilitating energy metabolism, and mitigating inflammatory responses [13]. Metabolomics allows a comprehensive analysis of the metabolic profiles of organisms, revealing alterations in the overall metabolic system. A study on systemic autoimmune diseases (SADs), including systemic lupus erythematosus, SS, and primary antiphospholipid syndrome, demonstrated a strong association between intestinal microflora and metabolic function in patients with SAD and highlighted specific metabolomic characteristics [19].

Currently, there are no studies on the mechanisms of action of TAIII in SS treatment. In this study, we used *16S* rRNA sequencing, SCFA analysis, and a non-targeted metabolomics approach to investigate the effects of TAIII on SS model mice.

## Materials and methods

### Materials

TAIII (CAS number: 41059–79-4; Sigma, St. Louis, MO, USA); hydroxychloroquine (HCQ; batch number: H19990263; Shanghai Pharmaceutical Company); mouse TNF-α (batch number: GR3200683-4) and mouse IL-17A (batch number: GR2906751-3) ELISA kits (Abcam, Cambridge, UK); anti-AQP1 and anti-AQP5 antibodies (item numbers: AB2219, AB15858; Sigma); methanol (catalog no. A452-4); and acetonitrile (catalog No. A998-4) and formic acid (catalog no. A117-50; Thermo Fisher Scientific, Waltham, MA, USA) were used.

### Animal experiments

Non-obese diabetic (NOD) mice were used to construct the SS disease model [20]. Twelve SPF-grade female BALB/c (8 weeks old) and 36 SPF-grade female NOD mice (8 weeks old) were procured from Beijing Huafukang Biotechnology Co., Ltd. (license no. SCXK (Beijing) 2019–0008). Throughout the experimental phase, the mice were housed by the Animal Experimentation Center of Guang’anmen Hospital of China Academy of Traditional Chinese Medicine, with six mice per cage. The control group consisted of 12 BALB/c mice. NOD mice were randomly divided into three groups based on body weight: the model, TAIII, and HCQ groups, with 12 mice in each group. TAIII or HCQ was continuously administered to each group by gavage from 9 weeks of age. The model group received an equal volume of deionized water once daily for 4 weeks until the end of the experiment. The clinical dose of HCQ was 400 mg/day for adults (60 kg), equivalent to 6.67 mg/(kg·day), which was converted to a gavage dose of 1.2 mg/(kg·day) for the mice. TAIII-treated mice were administered 30 mg/(kg·day) TAIII for four consecutive weeks. Fecal samples were collected at 4-week intervals, divided into 1-g aliquots, frozen within 4 h of collection, and stored at − 80 °C until subsequent analysis. This study was reviewed and approved by Medical Ethics Committee of Guang’anmen Hospital of China Academy of Traditional Chinese Medicine (2024–109-KY).

### Histopathological analysis

The salivary glands of each mouse were collected for histopathological analysis and stained with hematoxylin and eosin (HE). Images were captured using an FSX100 microscope (Olympus Corporation, Tokyo, Japan). Three experienced pathologists were asked to score them according to the Cutzler method, and the scores for each parameter ranged from 0 to 4. The higher the score, the greater the severity of the salivary gland tissue damage.

### ELISA of serum TNF-α and IL-17 levels

TNF-α and IL-17 serum levels in mice were measured using a bispecific antibody sandwich ELISA, following the manufacturer’s instructions. Standards (50 µL) at various gradient concentrations, along with serum samples diluted by fivefold, were added to plates pre-coated with TNF-α and IL-17 antibodies. Subsequently, 100 μL of horseradish peroxidase-labeled detection antibody was added to each well and incubated at 37 °C for 1 h, followed by five washes. Next, 50 μL of chromogenic substrates A and B were added to each well and were incubated at 37 °C, away from light. When a distinct blue gradient appeared in the standard wells, a stop solution was added, and the optical density (OD) was immediately measured at a wavelength of 450 nm. TNF-α and IL-17 concentrations were positively correlated with the OD values, and the amounts of TNF-α and IL-17 in the samples were calculated using the standard curve equation and dilution factor.

### Western blotting analysis of APQ1 and APQ5 protein expression in mouse submandibular gland tissues

Mouse submandibular gland tissue samples were weighed, and RIPA lysis buffer containing 1% PMSF was added. Tissues were cut, lysed on ice for 30 min, and centrifuged at 12,000 rpm (centrifugation radius of 8 cm) for 10 min. The supernatant was collected, and protein concentration was quantified using BCA assay. Proteins were isolated, separated using sodium dodecyl sulfate–polyacrylamide gel electrophoresis, and transferred to PVDF membranes. A 5% skimmed milk powder solution was added for 2 h at room temperature. Anti-AQP1 (1:1,000) and anti-AQP5 (1:1,000) antibodies were added and incubated overnight at 4 °C. After washing, a secondary antibody (1:5000) was added, and the membrane was incubated at room temperature for 2 h. ECL detection was performed, and the images were scanned. Image Lab software was used to analyze the gray values of the protein bands, with *β*-actin serving as an internal control for the calculation of the relative expression levels of the proteins of interest.

### Fecal DNA extraction and 16S rRNA sequencing

Bacterial DNA was extracted using the DNeasy PowerSoil kit (Qiagen, Germany). DNA concentration and integrity were assessed using NanoDrop 2000 (Thermo Fisher Scientific) and agarose gel electrophoresis. The V3–V4 regions of the bacterial *16S* rRNA gene were amplified in a 25-μL reaction solution with universal primers (343F: 5′-TACGGRAGGCAGCAG-3′, 798R: 5′-AGGGTATCTAATCCT-3′). Amplicon quality was determined using gel electrophoresis, and the PCR products were purified using Agencourt AMPure XP beads (Beckman Coulter, USA). Quantification was performed using a Qubit dsDNA assay kit, and the concentrations were adjusted for sequencing. Sequencing was performed using an Illumina NovaSeq6000 with two paired-end read cycles with 250 bases each. Raw data in the FASTQ format were preprocessed with “cut adapters” to remove adapters, followed by filtering, denoising, merging, and chimera removal using DADA2 with default QIIME2 parameters.

### Metabolomics analysis

Samples stored at − 80 °C were thawed at 27 °C, and 50 mg of the sample was mixed with 100 μL L-2-chlorophenylalanine in methanol (internal standard). After vortexing, 100 μL ice-cold methanol–acetonitrile mix was added, followed by ultrasonication and storage at − 20 °C. After centrifugation at 4 °C, 100 μL of supernatant was freeze-dried, reconstituted in a methanol–water mix, vortexed, ultrasonicated, and stored again at − 20 °C. After the final centrifugation step, the supernatant was filtered and transferred to LC vials for storage at − 80 °C until liquid chromatography–mass spectrometry (LC–MS) analysis. QC samples were pooled from the aliquots. The analysis was performed using a Dionex UHPLC system with a Q-Exactive mass spectrometer (Thermo Fisher Scientific). An ACQUITY UPLC HSS T3 column (1.8 μm, 2.1 × 100 mm) was employed in both positive and negative modes. The binary gradient elution system consisted of (A) water (containing 0.1% formic acid, v/v) and (B) acetonitrile (containing 0.1% formic acid, v/v), and separation was achieved using the following gradient: 0.01 min, 5% B; 2 min, 5% B; 4 min, 30% B; 8 min, 50% B; 10 min, 80% B; 14 min, 100% B; 15 min, 100% B; 15 min, 5% B; and 18 min, 5% B. The flow rate was 0.35 mL/min at 45 °C, with an injection volume of 5 μL. The mass range was 100–1,000 m/z, with a resolution of 70,000 for MS and 17,500 for higher energy collisional dissociation MS/MS. LC–MS data were processed using Progenesis QI, and metabolites were identified using the HMDB, Lipid Maps, and Metlin databases. Partial least squares discriminant analysis (PLS-DA) was performed in R using cross-validation and permutation testing. Significantly differential metabolites were identified using |log2FC|> 0.58 and *p* < 0.05, and pathway analysis was conducted using MetaboAnalyst.

### Statistical analysis

The nonparametric Kruskal–Wallis test was used to measure the differences between the two groups. Results are shown as the median ± IQR; significance was set at *p* < 0.05.

## Results

### TAIII effectively alleviated dry mouth in NOD mice

**Fig. 1:**
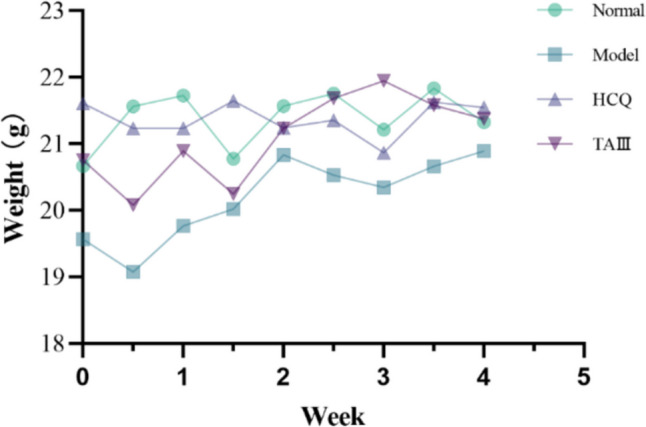
Differences in weight changes in mice between four groups during the experiment

Prior to the experiment, each group was acclimated for one week. On the first day of drug treatment, each mouse was weighed. Subsequently, the weight was recorded twice per week throughout the experiment. A line chart of the weight changes of the mice in each group was generated (Fig. 1). The weights of the mice remained within the normal range, with the average weight between 19 and 22 g. Additionally, the coat color was vibrant, and the overall mental state of the mice appeared normal.

**Fig. 2:**
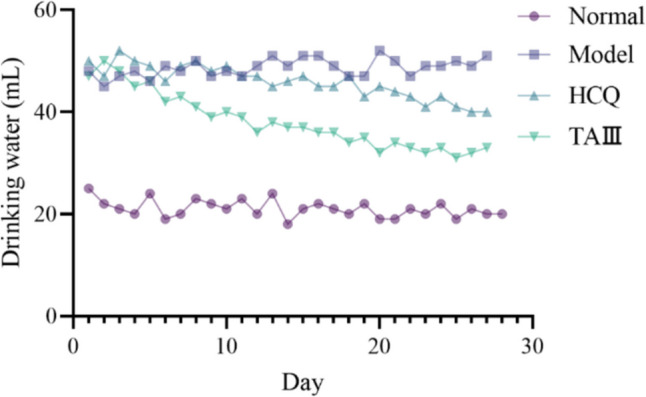
Changes in water intake of mice in the four groups during the experiment

The water intake of mice in the control and model groups aligned with their respective physiological characteristics. Specifically, the daily water intake of the control group ranged from 20 to 30 mL, whereas that of the model group was 45–55 mL, which was attributed to the dry mouth condition experienced by the mice. Following treatment with HCQ and TAIII, the daily water intake of mice decreased in both groups, suggesting that HCQ and TAIII enhance taste perception in NOD mice. Notably, the line chart (Fig. 2) revealed that the water intake of mice in the TAIII group decreased more rapidly. Furthermore, the daily water intake of the TAIII group gradually stabilized during the middle and later stages of the experiment, indicating that TAIII has significant advantages over HCQ in alleviating dry mouth in NOD mice.

### TAIII improved salivary gland tissue damage

**Fig. 3:**
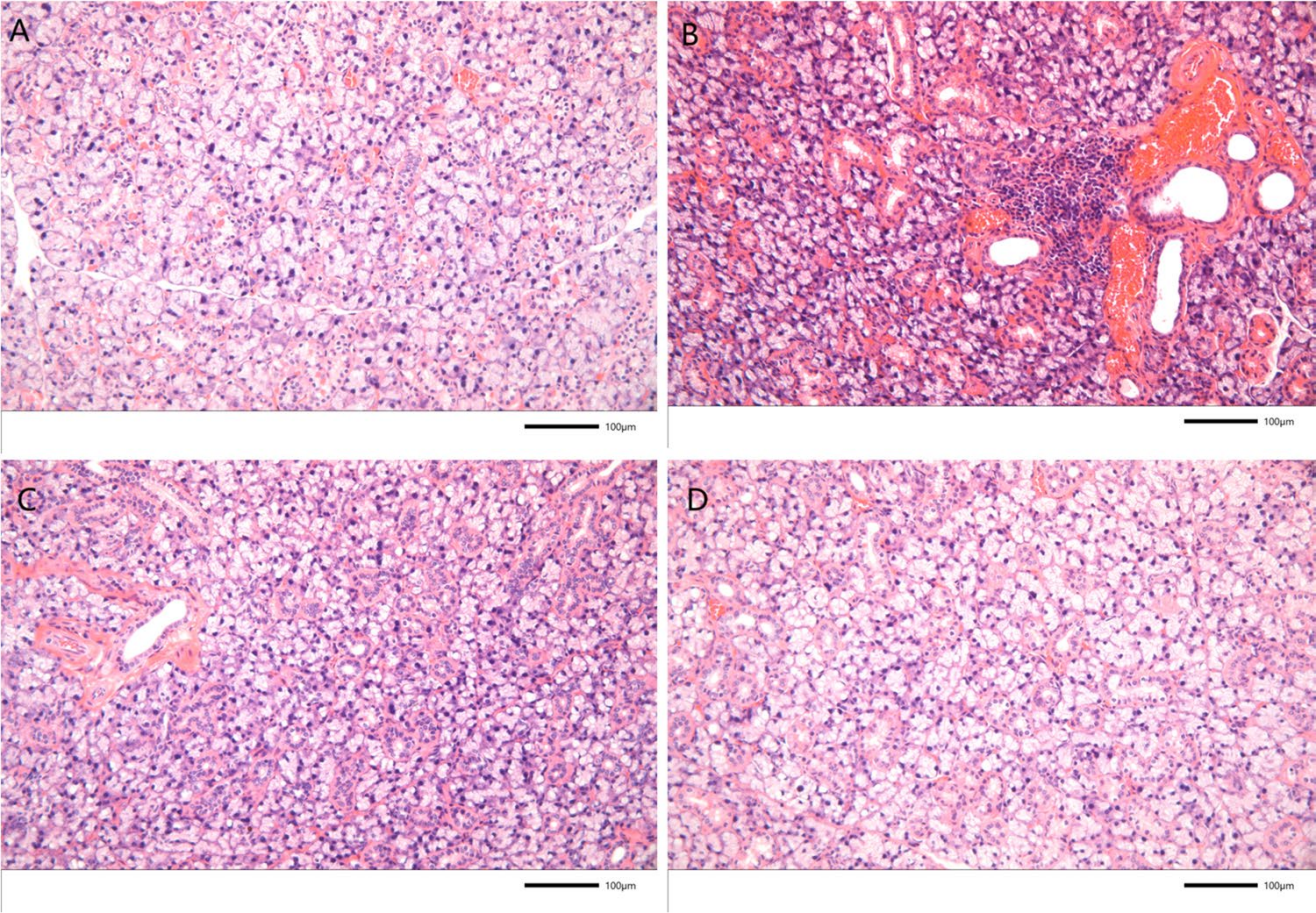
HE staining of the mice salivary gland tissue. (**A**) Control group. (**B**) Model group. (**C**) HCQ group. (**D**) TAIII group

HE staining showed that in the control group, the submandibular acinar and duct structures were normal, the acinar size remained consistent, and there was no lymphocyte infiltration into the interstitium (Fig. 3A). In the model group, lymphocytes significantly infiltrated into the interstitium. The size of the acini varied, and some were destroyed. Additionally, local glandular atrophy and proliferation of fibrous tissue were observed (Fig. 3B). Moderate lymphocytic infiltration was observed in the HCQ group, along with local gland atrophy and hyperplasia of the fibrous tissue (Fig. 3C). However, the pathological manifestations of the submandibular gland in the TAIII group exhibited a minimal amount of scattered single-lymphocyte infiltration, with variations in acinar size and occasional destruction of a small number of acinar and ductal structures (Fig. 3D). These results indicated that TAIII can relieve salivary gland tissue damage.

The pathological score for the salivary gland tissue in the model group was significantly higher than that in the control group (*p* < 0.001). Compared with that of the model group, the histopathological score of the salivary gland in the TAIII group was significantly lower (*p < *0.01) (Table 1).

**Table 1:** The mean pathological score of mouse salivary glands in each experimental group

Group	*N*	Pathological score
Control	12	0.00 ± 0.00***
Model	12	3.89 ± 0.48
Hydroxychloroquine	12	2.33 ± 0.52**
Timosaponin AIII	12	1.50 ± 0.33**

Data are expressed as mean ± SD; *n* = 12

*p < 0.05, **p < 0.01, ***p < 0.001 vs. model group

### TAIII reduced the expression of serum inflammatory factors

**Fig. 4:**
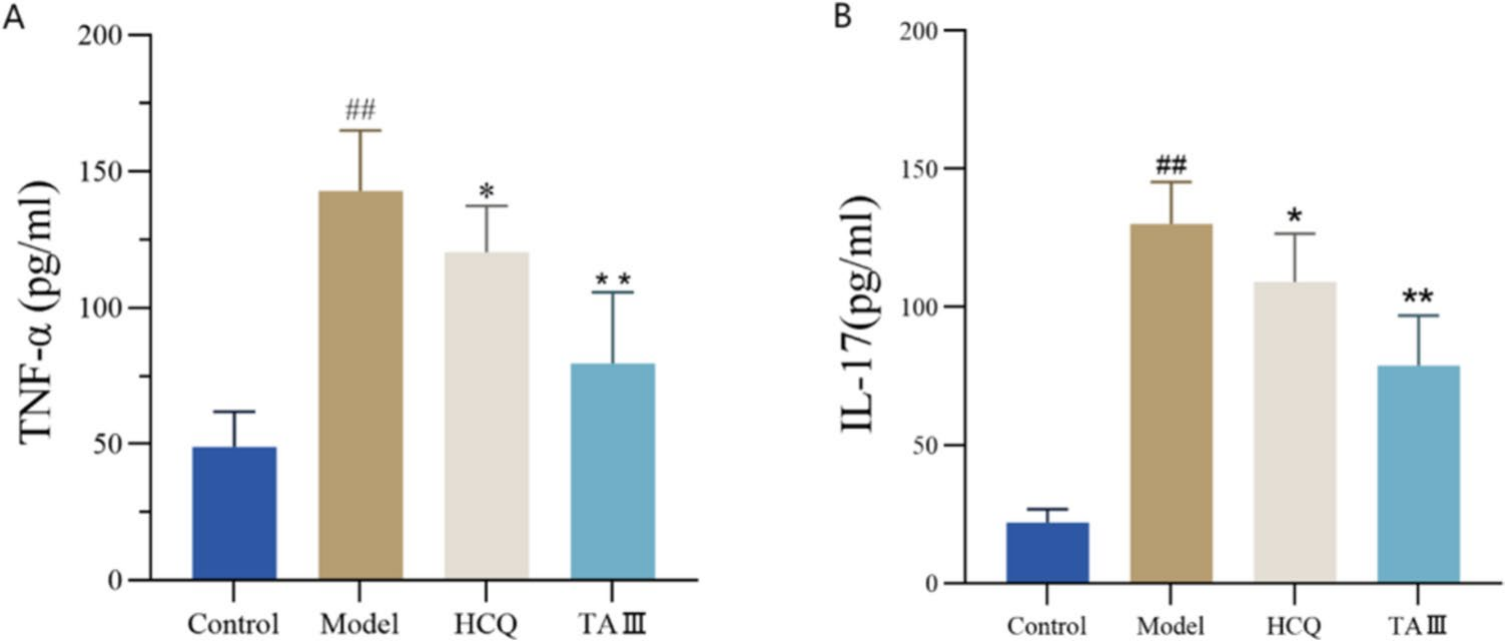
Effect of TAIII on the expression of serum inflammatory factors in NOD mice. (**A**) TNF-α. (**B**) IL-17. *p < 0.05, **p < 0.01 vs. Model group

The ELISA results indicated that the expression of TNF-α and IL-17 in the model group was significantly higher than that in the control group (*p* < 0.01). Following HCQ and TAIII treatment, TNF-α and IL-17 expression levels in the HCQ group significantly decreased compared with those in the model group (*p* < 0.05), whereas TNF-α and IL-17 expression levels in the TAIII group were the lowest (*p* < 0.01) (Fig. 4A, B). Therefore, TAIII effectively reduced the expression of inflammatory factors in NOD mice.

### TAIII increased the expression of AQP1 and AQP5

**Fig. 5:**
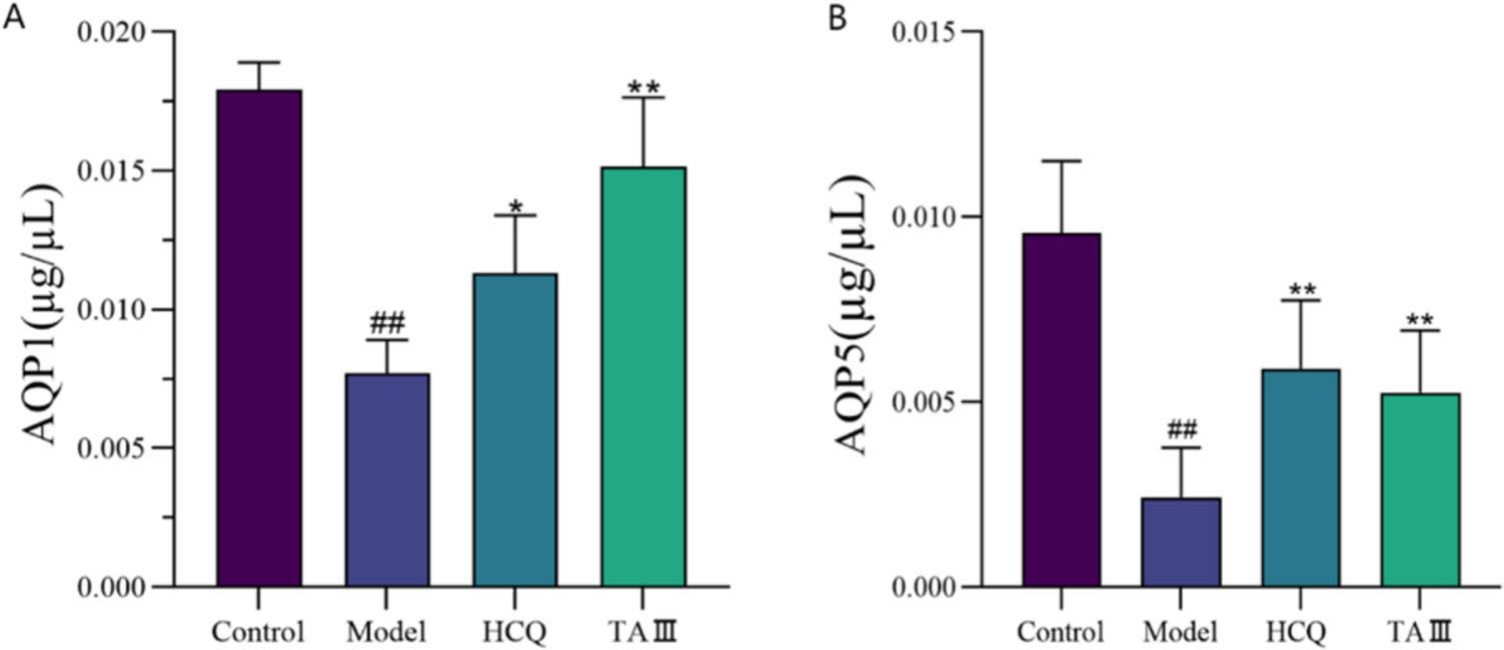
Effect of TAIII on the expression of AQP in NOD mice. (**A**) AQP1. (**B**) AQP5. *p < 0.05, **p < 0.01 vs. Model group

AQP1 and AQP5 expression levels in each group were assessed using western blotting. The results indicated that AQP1 and AQP5 expression levels in the model group were significantly lower than those in the control group (*p* < 0.01). AQP1 expression significantly increased in the HCQ group compared with that in the model group (*p* < 0.05). Additionally, TAIII treatment significantly enhanced AQP1 expression levels compared with those in the model group (*p* < 0.01), with the TAIII group showing a more pronounced effect than that in the HCQ group. AQP5 expression significantly increased in both the HCQ and TAIII groups (*p* < 0.01). Thus, TAIII may alleviate the disease state in NOD mice by modulating AQP1 and AQP5 expression (Fig. 5A, B).

### TAIII regulated gut microbiota dysbiosis in NOD mice

**Fig. 6:**
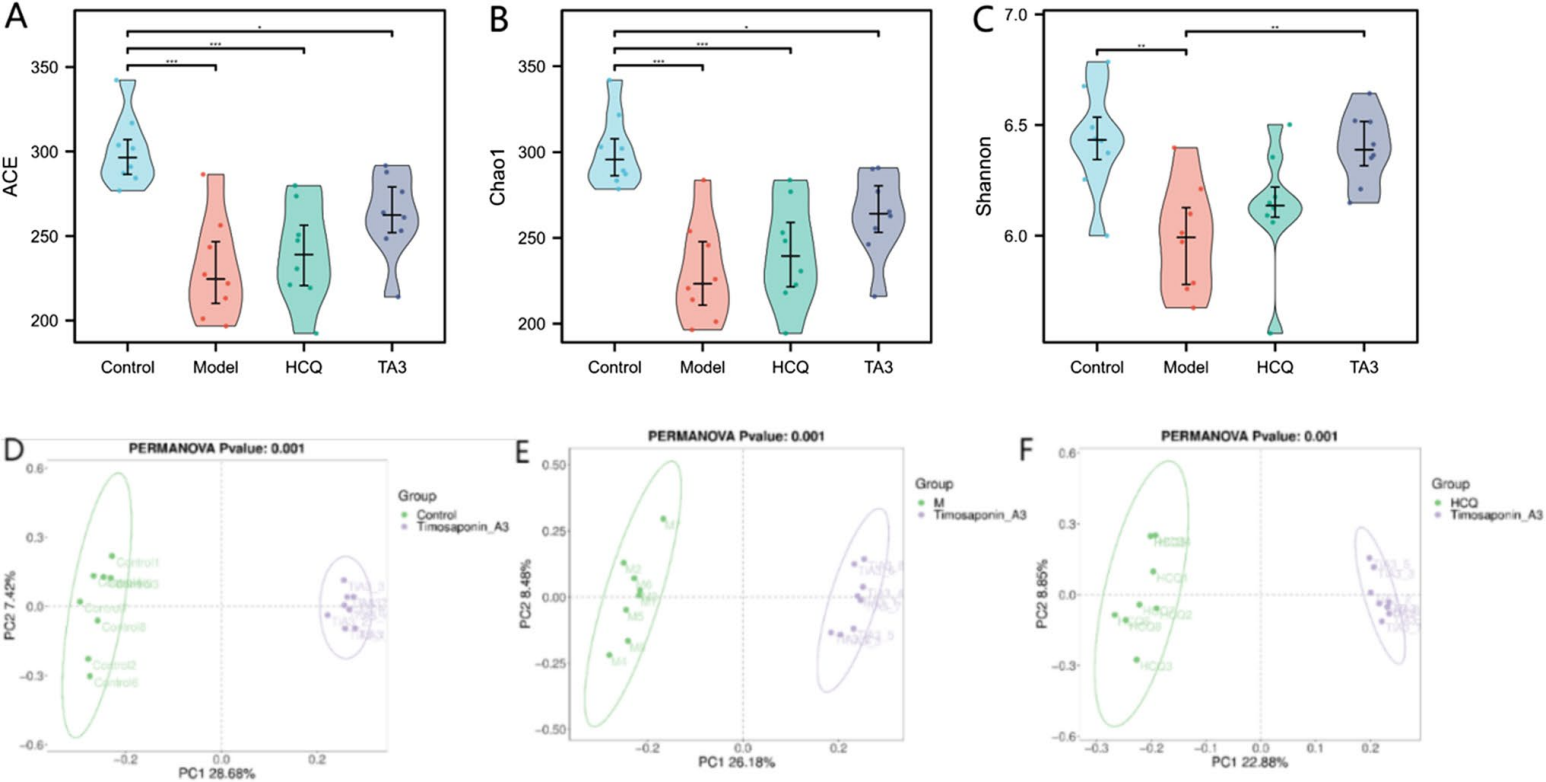
TAIII regulates gut microbial richness and diversity in NOD mice. (**A**) Ace index. (**B**) Chao index. (**C**) Shannon index. (**D**) Control-TAIII principal component (PCA) analysis. (**E**) Model-TAIII PCA. (**F**) HCQ-TAIII PCA. *p < 0.05, **p < 0.01

*16S* rRNA was sequenced and analyzed to determine the effect of TAIII on the gut microbiota dysbiosis in NOD mice. Alpha diversity analysis was performed to reveal the species abundance index; the ACE, Chao, and Shannon indices in the model group were significantly lower than those in the control group (*p* < 0.05). After TAIII administration, the species abundance in the HCQ and TAIII groups increased to varying degrees, with the increase in the TAIII group being more significant, and they subsequently returned to normal levels (Fig. 6A–C). Beta diversity analysis was performed to assess whether there were differences in microbiota composition between the different groups. Principal coordinate analysis indicated that there was a notable separation trend between the TAIII and other groups, and the groups were clearly distinguishable in the PLS-DA model (Fig. 6D–F). Therefore, TAIII improved gut microbiota diversity in NOD mice.

**Fig. 7:**
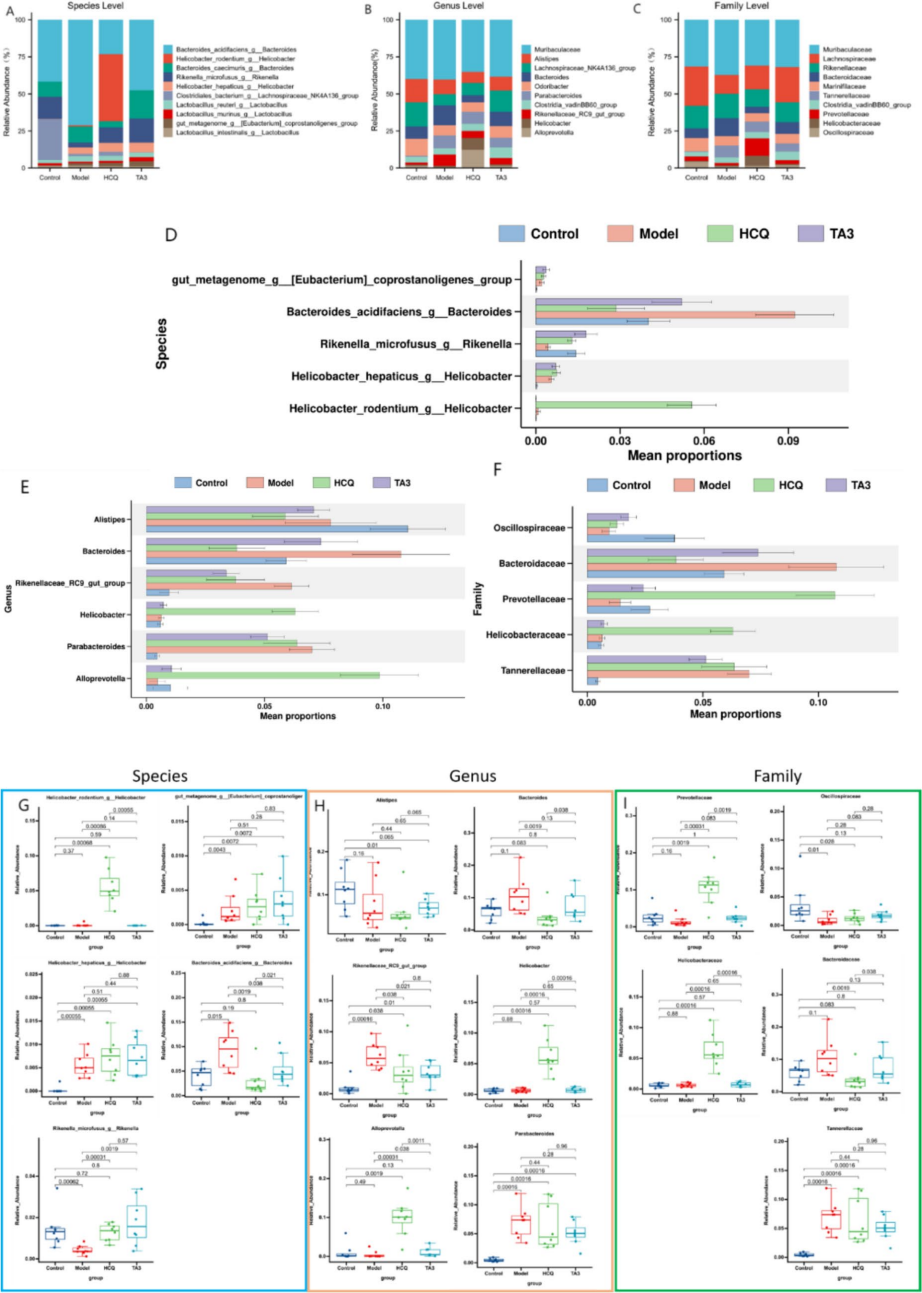
Gut microbial community structure in NOD mice after HCQ and TAIII treatment: (**A**) species level; (**B**) genus level; (**C**) family level. The difference of abundance at the species, genus, and family levels in the abundance analysis: (**D**) species level; (**E**) genus level; (**F**) family level. Regulation of key bacteria by TAIII and HCQ: (**G**) species level; (**H**) genus level; (**I**) family level. *p < 0.05, **p < 0.01, ***p < 0.001

**Fig. 8:**
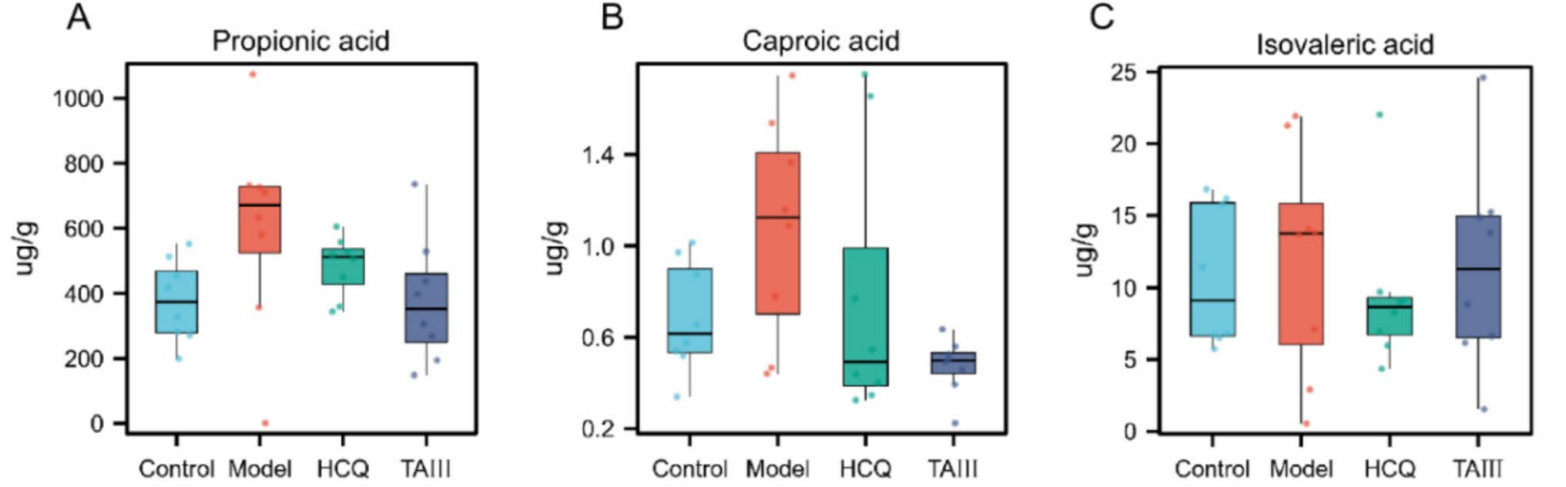
Treatment effects on SCFAs content in mice feces. (**A**) Propionic acid. (**B**) Caproic acid. (**C**) Isobutyric acid

The taxonomic composition of each group at the species, genus, and family levels was analyzed. The top 10 most abundant intestinal flora in each group at the species, genus, and family levels are illustrated in Fig. 7A–C. At the species level, the abundance of *Bacteroides_acidifaciens_g__Bacteroides, Lactobacillus_reuteri_g_Lactobacilus*, and *Lactobacillus_intestinals_g_Lactobacillus* increased in the model group, but decreased in the HCQ and TAIII groups, and the abundance of *Rikenella_microfusus_g__Rikenella* decreased in the model group and increased in the HCQ and TAIII groups. At the genus level, compared with those in the model group, the abundances of Lachnospiraceae NK4A136_group, *Odoribacter*, and *Alloprevotella* in the TAIII and HCQ groups increased, and the abundances of Muribaculaceae, *Bacteroides*, *Parabacteroides*, and Rikenellaceae_RC9_gut_group decreased. At the family level, the abundances of Muribaculaceae, Rikenellaceae, Bacteroidaceae, and Tannerellaceae in the HCQ and TAIII groups were lower than those in the model group, whereas the abundances of Lachnospiraceae, Marinifilaceae, and Oscillospiraceae were higher. Moreover, statistical analysis of the meta-genomic profiles was performed to screen out the significantly different species in the top 10 intestinal flora at the levels of species, genus, and family, as shown in Fig. 8. The relative abundance of the species differed significantly at the species, genus, and family levels, as shown in Fig. 7D–F. After TAIII and HCQ treatment, the abundances of *Rikenella_microfusus_g_Rikenella* and *Alloprevotella* significantly increased (*p* < 0.05), compared to those in the model group. However, the abundances of *Bacteroidesacidifaciens_g_Bacteroides* and Rikenellaceae _RC9_gut_group were significantly reduced (*p* < 0.05) (Fig. 7G–I).

### TAIII regulated SCFA levels in NOD mice

Concentrations of seven SCFAs (acetate, propionate, butyrate, valerate, caproate, isobutyrate, and isovalerate acid) in the feces of NOD mice were determined using gas chromatography–mass spectrometry analysis. Compared with the control group, the model group showed significantly increased concentrations of propionic, isovaleric, and caproic acid. After TAIII and HCQ treatment, the contents of propionic, isovaleric, and caproic acid exhibited a downward trend, although the difference was insignificant (*p* > 0.05) (Fig. 8A–C). Propionic acid levels significantly decreased after TAIII treatment, but then tended to return to normal. These results indicated that TAIII primarily improved the disease characteristics of NOD mice by regulating propionic acid levels.

### TAIII improved dysregulated fecal metabolite levels in NOD mice

**Fig. 9:**
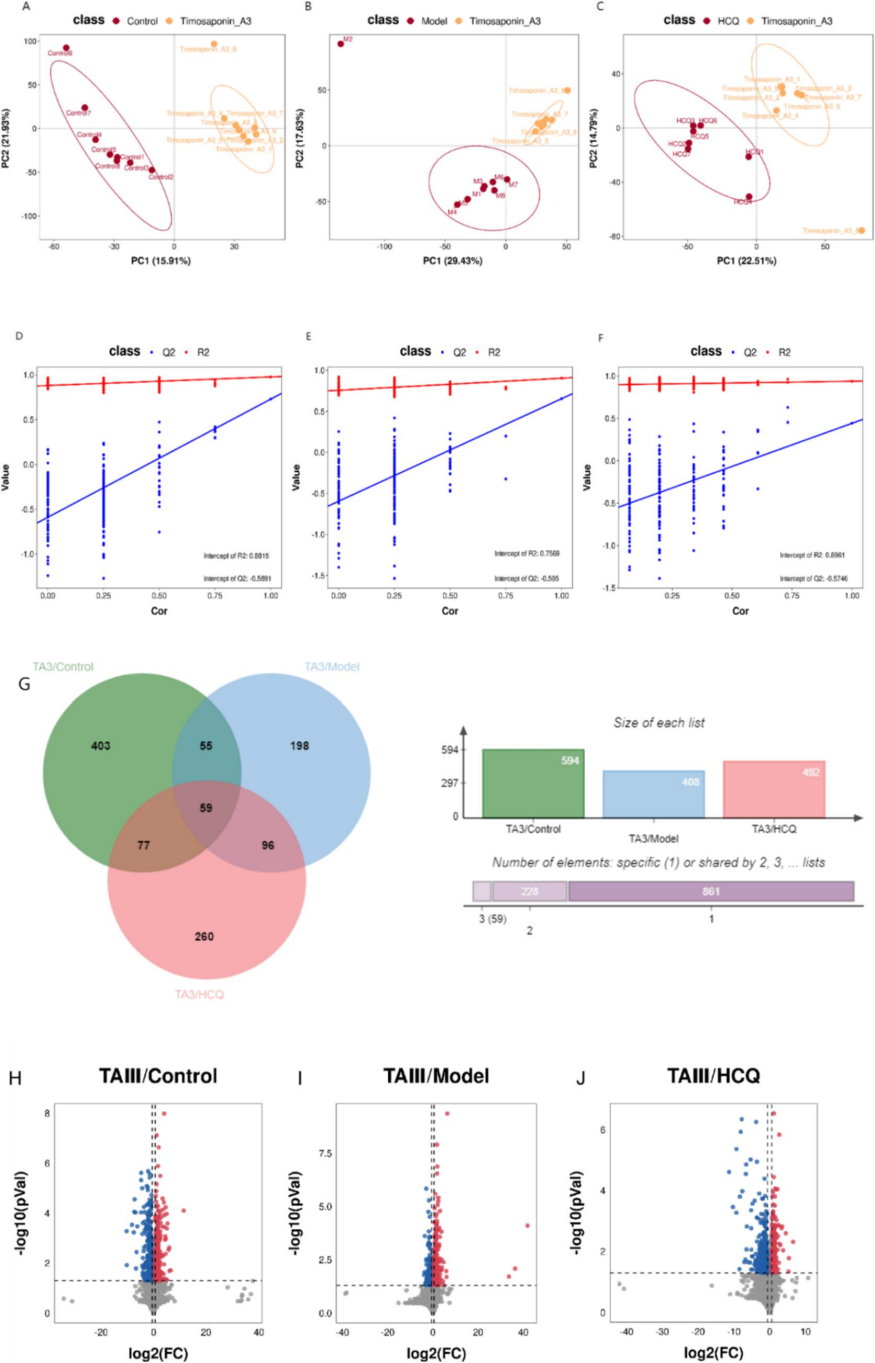

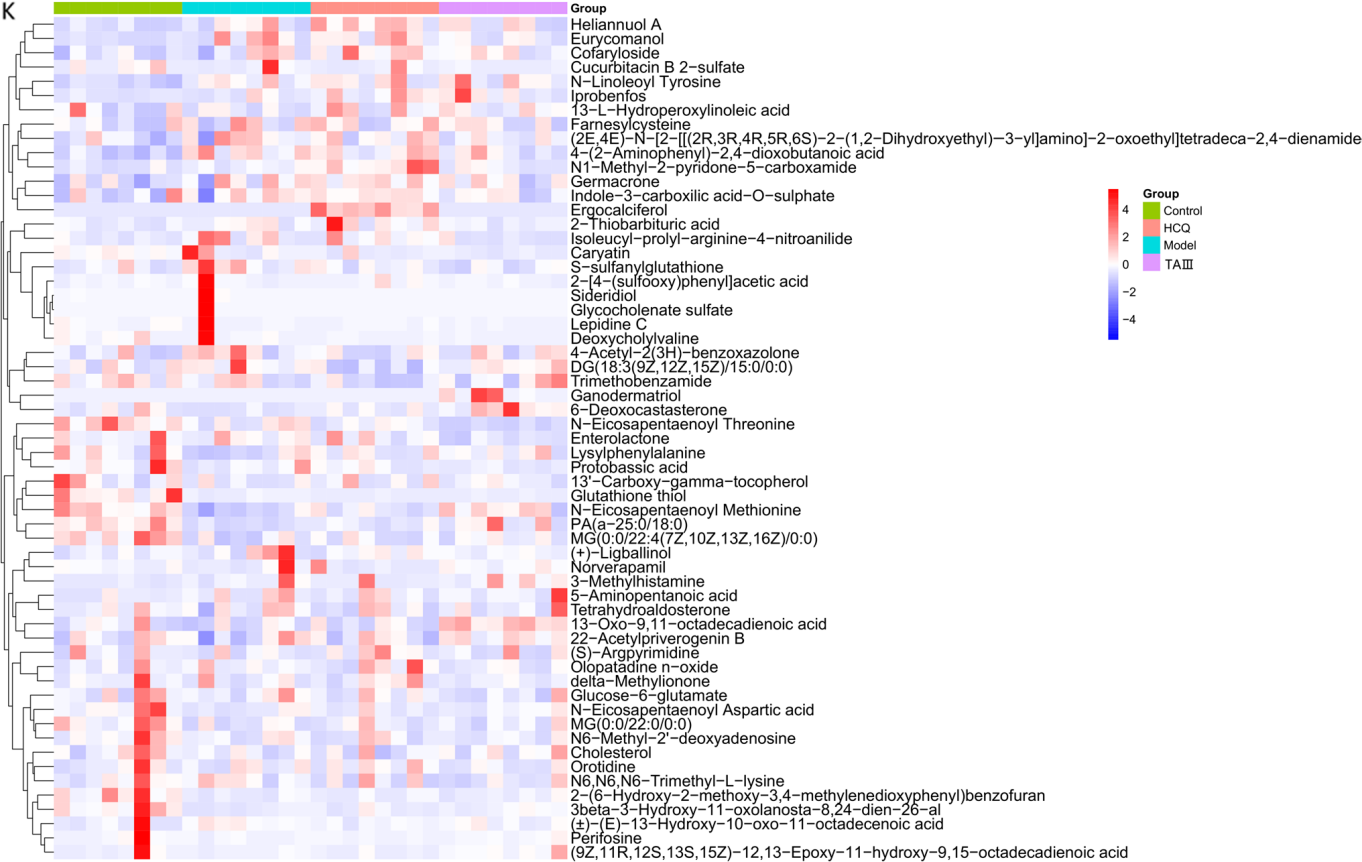
Effect of TAIII on fecal metabolites in NOD mice. The PLS-DA score: (**A**) Control vs. TAIII; (**B**) Model vs. TAIII; (**C**) HCQ vs. TAIII. Validation plot by permutation: (**D**) Control vs. TAIII; (**E**) Model vs. TAIII; (**F**) HCQ vs. TAIII. (**G**) Metabolites intersection union analysis of Venn diagrams. Metabolite differential volcano diagram: (**H**) Control vs. TAIII; (**I**) Model vs. TAIII; (**J**) HCQ vs. TAIII. (**K**) Differential metabolites heat map

Untargeted metabolomic analyses of mouse feces were performed using LC–MS to assess the effects of TAIII on gut microflora imbalance–related metabolic pathways. The PLS-DA score plots showed a significant difference in fecal metabolite levels between the TAIII and other groups (Fig. 9A–C). The comparisons yielded the following results: between the TAIII and control groups, *R*^2^ = 0.977 and *Q*^2^ = 0.729; between the TAIII and model groups, *R*^2^ = 0.905 and *Q*^2^ = 0.654; and between the TAIII and HCQ groups, *R*^2^ = 0.937 and *Q*^2^ = 0.441. The validated PLS-DA results showed that the model was stable and accurate (Fig. 9D–F).

Thirty-two fecal samples and three QC samples were analyzed using UHPLC-Q-TOF/MS. As shown in Fig. 9G, 2998 metabolites were identified in the ESI + and ESI − modes. Among these, the levels of 1148 metabolites differed between the TAIII and other groups (VIP > 1, ANOVA FDR < 0.05). Compared with the control group, the levels of 594 metabolites, including 282 upregulated and 312 downregulated metabolites, differed in the TAIII group. Compared with those in the model groups, the abundances of 408 metabolites differed in the TAIII group, comprising 257 upregulated and 151 downregulated metabolites. TAIII and HCQ group comparisons revealed 492 differentially abundant metabolites, with 193 upregulated and 299 downregulated metabolites. There were 59 differentially abundant metabolites among all the groups (Online Resource 1). A volcanic diagram illustrating the differential metabolites between the TAIII and other three groups is presented in Fig. 9H-J. A heatmap plot revealed 59 metabolites and their distributions (Fig. 9K). These metabolites were selected as references for further analysis.

**Fig. 10:**
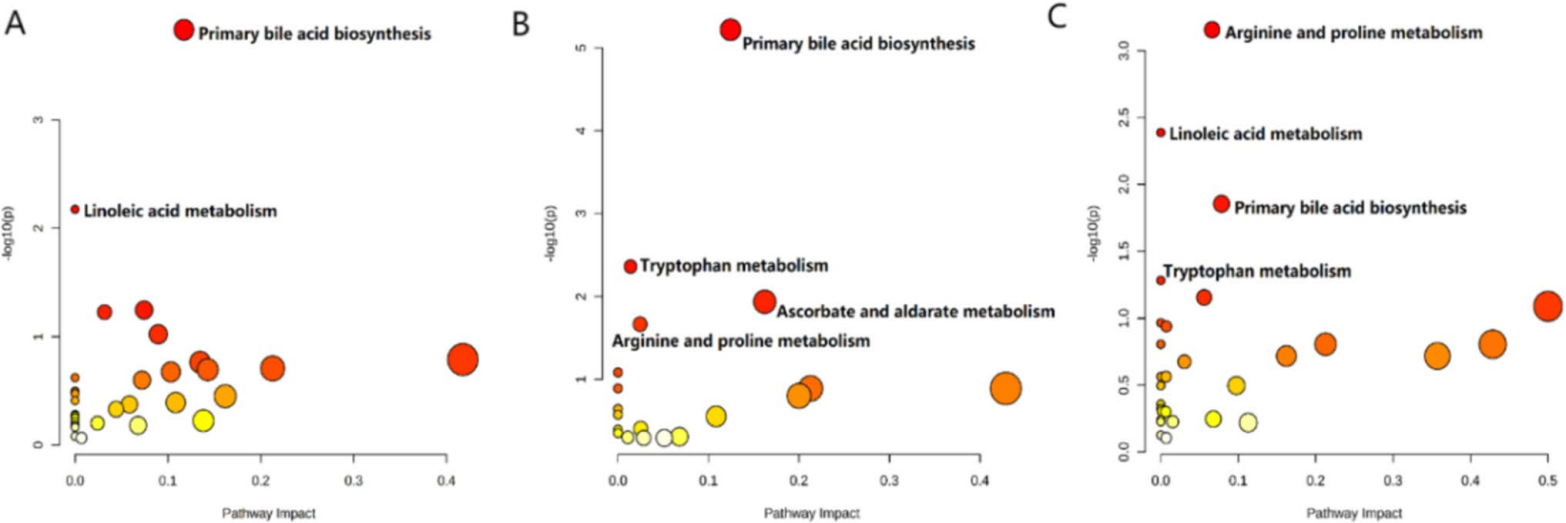
Key metabolic pathways regulated by TAIII: (**A**) Control vs. TAIII; (**B**) Model vs. TAIII; (**C**) HCQ vs. TAIII

KEGG pathway enrichment analysis was performed on the differentially abundant metabolites. Based on *p*-values and impact factors, the main metabolic pathways associated with the changes in metabolites between TAIII and the other groups were identified. Primary bile acid biosynthesis and linoleic acid metabolism pathways were significantly enriched in the TAIII and control group (*p* < 0.05). Compared with those in the model group, the predominant enriched metabolic pathways in the TAIII group were bile acid biosynthesis, tryptophan metabolism, arginine and proline metabolism, and ascorbate and aldarate metabolism (*p* < 0.05). Compared with those in the HCQ group, the key enriched pathways in the TAIII group were arginine and proline metabolism, linoleic acid metabolism, primary bile acid biosynthesis, and tryptophan metabolism (*p* < 0.05) (Fig. 10A–C).

### Correlation analysis between gut microbiota and metabolites

**Fig. 11:**
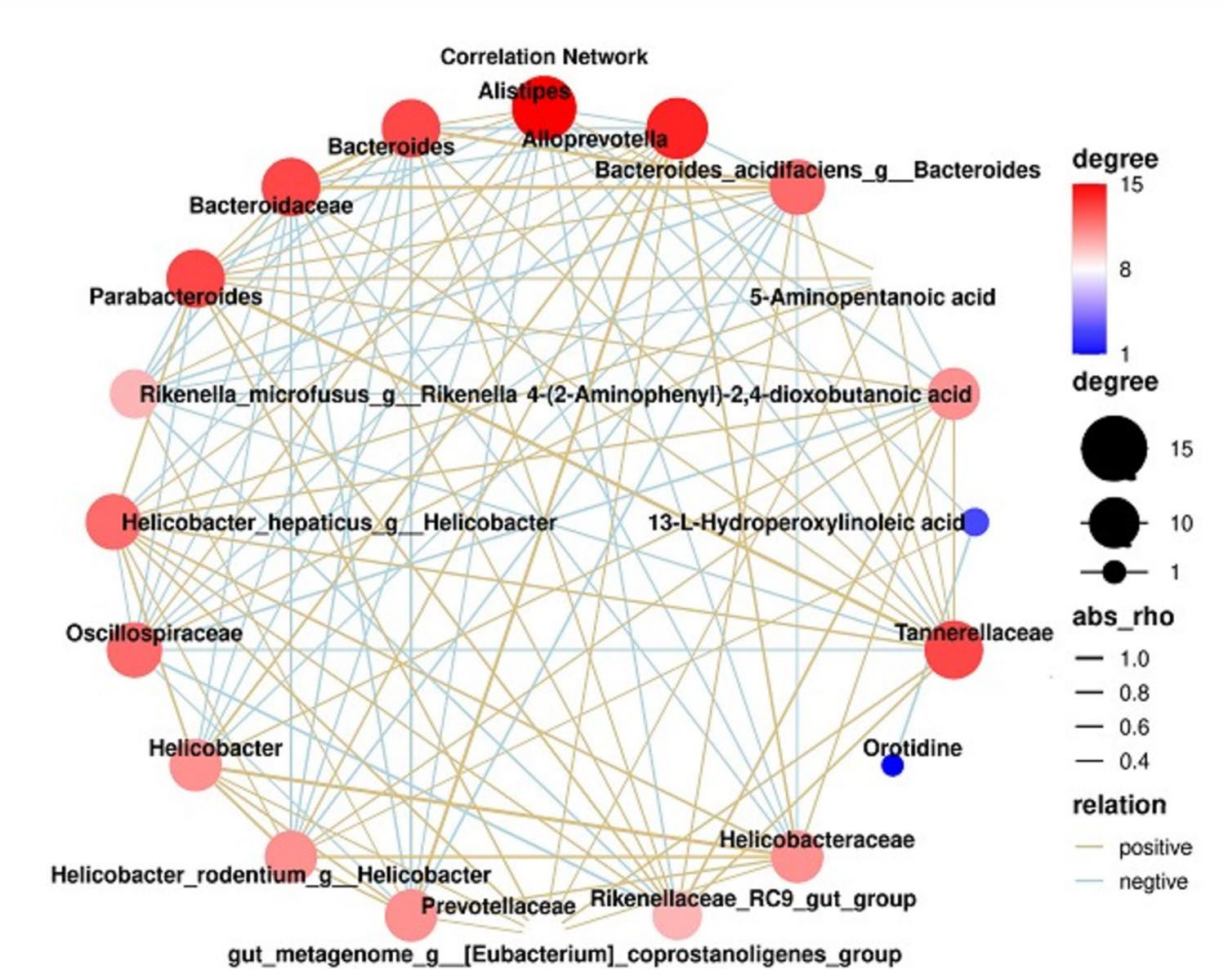
Correlation networks between key genera and key metabolites that show differential expression following TAIII action

To determine the relationship between gut microbiota and differential fecal metabolites, correlation analysis was conducted on all intestinal microorganisms (Fig. 7D–F) and on metabolic pathways (Fig. 9K) using Spearman correlation coefficients. The 4-(2-aminophenyl)−2,4-dioxobutanoic acid and 5-aminopentanoic acid levels were significantly correlated with the gut microbial community. Additionally, 4-(2-aminophenyl)−2,4-dioxobutanoic acid was significantly associated with eight bacterial groups: positively correlated with *Helicobacter hepaticus g__Helicobacter*, *Parabacteroides*, Rikenellaceae_RC9_gut_group, *Helicobacter*, Tannerellaceae, and Helicobacteraceae; and negatively correlated with *Alistipes* and Oscillospiraceae. The 5-aminopentanoic acid was closely positively correlated with Helicobacter hepaticus and *Alistipes* (Fig. 11). The 4-(2-aminophenyl)−2,4-dioxobutanoic acid was found to be a metabolite of the tryptophan metabolic pathway, and 5-aminopentanoic acid was a metabolite of the arginine and proline metabolic pathways. TAIII significantly regulated the abundance of Rikenellaceae_RC9_gut_group (*p* < 0.05), which subsequently returned to normal levels. The Rikenellaceae_RC9_gut_group was a key bacterium and tryptophan metabolism was the main metabolic pathway.

## Discussion

In this study, we used *16S* rRNA sequences and non-target metabolism to explore the therapeutic mechanism of TAIII in SS and found that the Rikenellaceae_RC9_gut_group was the most important intestinal bacteria and tryptophan metabolism was the main metabolic pathway involved. Therefore, we believe that TAIII treats SS by regulating the intestinal microbial community structure and metabolite levels.

SS pathogenesis involves inflammatory cytokine production and lymphocyte infiltration [7]. Inflammatory factors, such as IL-17 and TNF-α, play a crucial role in the development of pathological changes and lymphocyte-mediated tissue damage. IL-17 expression was significantly elevated in SS mouse models, which contributes to lymphocyte infiltration in salivary glands and lesion formation [21, 22]. Additionally, IL-17 protein and mRNA levels in the peripheral blood of patients with SS are increased. TNF-α serves as a potent regulator of inflammation and autoimmune diseases [23]. It mediates inflammation by activating the NF-kB and MAPK pathways [24]. Activated macrophages in patients with SS produce inflammatory cytokines, such as TNF-α, which can lead to cellular damage [25, 26]. In this study, we demonstrated that TAIII effectively reduces the expression of IL-17 and TNF-α in NOD mice.

AQP1 and AQP5 are major aquaporins that play important roles in salivation [27]. Abnormal expression and localization of AQP5 lead to xerostomia in SS [28]. AQP1 expression is downregulated in the salivary glands of patients with SS [29], and the elimination of B cells with rituximab can increase the expression of AQP1 in the myoepithelial cells of patients with SS, as well as the saliva flow rate [30]. The localization of AQP5 on the apical side of patients with SS is reduced, which affects salivary secretion [31]. In this study, western blotting revealed that TAIII significantly upregulated AQP1 and AQP5 expression in NOD mice.

The intestinal microflora plays a crucial role in maintaining intestinal homeostasis and has considerable potential for regulating human immune function [13]. Changes in the intestinal microflora can affect immune system functionality [14]. Furthermore, the severity of SS is linked to systemic disease activity and gastrointestinal inflammation [32]. The Rikenellaceae_RC9_gut_group, of the Bacteroidetes phylum, primarily contributes to the degradation of fiber and other complex carbohydrates, and is closely associated with intestinal immune regulation. In a high-fat diet-induced metabolic disorder model, the abundance of Rikenellaceae_RC9_gut_group increased, suggesting its potential role in inflammation and metabolic disorders. Following the intervention, its abundance significantly decreased [33]. In another study, mice treated with probiotics showed a notable reduction in the abundance of the Rikenellaceae_RC9_gut_group [34]. Pentachlorophenol is a carcinogenic substance widely used as a preservative and disinfectant, and its intervention leads to a higher abundance of Rikenellaceae_RC9_gut_group in mice with colitis [35]. Resveratrol is a natural plant antioxidant polyphenol with anti-inflammatory properties. After adding resveratrol to the feed, it was found that the levels of Rikenellaceae_RC9_gut_group in the ewes’ bodies changed significantly, suggesting that Rikenellaceae_RC9_gut_group is related to inflammation and immunity [36]. This suggests that under an imbalance in the intestinal flora, an increase in the abundance of Rikenellaceae_RC9_gut_group may be linked to disease progression. In this study, the abundance of Rikenellaceae_RC9_gut_group significantly increased in NOD mice. After TAIII intervention, there was a significant reduction in its abundance, which helped to restore the balance of the intestinal flora.

Non-digestible carbohydrates are transformed into SCFAs by specific intestinal microbes [37]. SCFAs can alleviate inflammatory bowel disease by suppressing the release of pro-inflammatory factors [38]. In this study, the effects of SCFAs were assessed after TAIII administration. The findings suggested that SCFAs, particularly propionic acid, play a role in reducing inflammation and suppressing SS. Specifically, propionic acid triggers a pro-inflammatory response, primarily by activating the MAPK signaling pathway, which leads to the production of inflammatory cytokines [39]. Therefore, TAIII may improve SS by regulating the levels of SCFAs, such as propionic acid. The Rikenellaceae_RC9_gut_group is primarily involved in the degradation of cellulose and polysaccharides, and its metabolic activity promotes the production of propionic acid, consistent with a previous study [40]. Research had shown that the Rikenellaceae_RC9_gut_group is involved in the digestion and absorption of nutrients, breaking down carbohydrates into SCFAs [41], and influencing the levels of metabolic products [36]. Furthermore, the Rikenellaceae_RC9_gut_group is negatively correlated with the levels of anti-inflammatory factors, such as S-glutaryldihydrolipoamide, which exacerbate inflammatory responses by influencing the metabolic processes involving propionic acid [42]. TAIII may decrease the abundance of Rikenellaceae_RC9_gut_group and propionic acid levels, thereby reducing the inflammatory response in NOD mice.

Gut bacteria influence host metabolism by releasing small molecules into the circulatory system [43]. The levels of 4-(2-aminophenyl)−2,4-dioxobutanoic acid are significantly increased in various diseases. Another study demonstrated that in patients with type 1 diabetes mellitus, certain metabolic levels markedly increased, but significantly decreased following intervention. Moreover, 4-(2-aminophenyl)−2,4-dioxobutanoic acid may serve as a potential biomarker for predicting the progression of metabolic disorders [44]. Another study found that 4-(2-aminophenyl)−2,4-dioxobutanoic acid levels, which is associated with inflammation [45], are increased in patients with diarrhea-predominant irritable bowel syndrome, but significantly reduced after probiotic intervention. As a marker of oxidative stress and inflammation, elevation of its levels correlates with a decline in function, immune disorders, and alterations in the intestinal microbiota [46]. A decrease in its levels can be achieved through metabolic interventions or pharmacological treatments, suggesting that metabolic pathways may play a role in disease management. In this study, 4-(2-aminophenyl)−2,4-dioxobutanoic acid was identified as an important metabolite of the therapeutic effects of TAIII in SS and was positively correlated with the abundance of Rikenellaceae_RC9_gut_group. Therefore, TAIII may enhance the inflammatory response in NOD mice by modulating metabolite levels. The main metabolite involved in tryptophan metabolism is 4-(2-aminophenyl)−2,4-dioxobutanoic acid. In intestinal microorganisms, tryptophan is metabolized into indole and its derivatives, such as indole-3-acetic acid and indole-3-propionic acid. The Rikenellaceae_RC9_gut_group plays a role in tryptophan metabolism and produces indole derivatives, particularly indole-3-acetic acid, which regulates the host’s immune and metabolic functions and the inflammatory response through the aromatic hydrocarbon receptor pathway [47, 48]. In this study, TAIII regulated tryptophan metabolism through the Rikenellaceae_RC9_gut_group, modulated immune function and inflammatory responses in NOD mice, and alleviated SS symptoms. This study elucidates the regulatory mechanisms of TAIII in immune responses and metabolic disorders, revealing its potential role in SS treatment. Although this study has limitations in sample size and the lack of serum metabolite testing, it provides valuable references for the targeted treatment of SS with traditional Chinese medicine.

TAIII can effectively improve the disease state of NOD mice and offers a novel approach for the prevention and treatment of SS. TAIII not only inhibits excessive inflammatory responses in the intestine but also restores the homeostasis of intestinal flora by regulating microbial metabolites and metabolic pathways. This function was primarily achieved by TAIII through the modulation of the abundance of the Rikenellaceae_RC9_gut_group, and SCFA and tryptophan levels, which are new potential targets for TAIII in SS treatment. From the perspective of intestinal flora, SCFAs, and metabolomics, this study provides a new foundation for further understanding the potential mechanisms by which TAIII alleviates SS, facilitating drug development and clinical treatment.

## Supplementary Information

Below is the link to the electronic supplementary material.

Supplementary file1 (XLSX 52 KB)

## Data Availability

Our research data can be found in the article and supplementary materials.
